# Manifestations of verbal and physical violence towards doctors: a comparison between hospital and community doctors

**DOI:** 10.1186/s12913-019-4700-2

**Published:** 2019-11-26

**Authors:** Tamar Nevo, Roni Peleg, Daniel M. Kaplan, Tamar Freud

**Affiliations:** 1 Division of Community Health, Faculty of Health Sciences, Ben-Gurion University of the Negev, P.O. Box 653, 84105 Beer-Sheva, Israel; 2 Siaal Research Center for Family Medicine and Primary Care, Department of Family Medicine, Division of Health in the Community, Faculty of Health Sciences, Ben-Gurion University of the Negev, Beer-Sheva, Israel; 3 Clalit Health Services, Beer-Sheva, Southern District Israel; 4 Department of Otolaryngology- Head and Neck Surgery, Soroka University Medical Center, Beer-Sheva, Israel

**Keywords:** Hospital doctors, Community doctors, Violence, Physical abuse, Verbal abuse

## Abstract

**Background:**

Healthcare workers, in the hospital and in community clinics, are frequently exposed to verbal and physical abuse that can lead to frustration and despair. This study’s objectives were to evaluate trends in violence towards hospital and community doctors in the Negev region of Israel and to compare them to the results of a previous 2005 study.

**Methods:**

A convenience sample of doctors in the hospital and in the community completed anonymous questionnaires on previous exposure to work place violence and their attitudes to it. The data were collected in 2017.

**Results:**

One hundred forty-five doctors participated in the study, of who 63 were hospital doctors and 82 were community doctors. Fifty nine percent of the doctors reported that they experienced at least one incident of verbal abuse over the previous year and 9% were exposed to physical abuse, compared to 56 and 9%, respectively, in the previous study. More hospital doctors (58.7%) were exposed to verbal abuse on the part of family members than community doctors (35.8%) (*P* = 0.007). The most common reason for a violent outbreak was long waiting times, followed by dissatisfaction with treatment, both consistent with the findings in the previous study. Seventy one percent said that violence was a major problem for doctors. The majority (73.9%) had not participated in a workshop or other training for preventing workplace violence or coping with it, an improvement over the 83% who reported not receiving any training in the previous study.

**Conclusions:**

Workplace violence is a major issue, which affects hospital and community physicians alike. There is a rise in the number of doctors who have undergone training in this area, although the majority have yet to receive formal training.

## Background

Over recent years there has been an increase in acts of violence in Israel, as in the rest of the world. Violence has increased in the workplace, in places of entertainment, in schools, on highways, and in cultural institutions. The media constantly reports on acts of violence. Violence is not necessarily physical. It can also be verbal or mental. Sometimes, emotional torment or verbal attacks can lead to more severe outcomes than physical abuse. Healthcare workers in hospital and community clinics are exposed frequently to verbal or physical abuse, which can lead to frustration and despair [1–4].

Doctors often are a target for workplace violence. About a quarter of emergency room doctors reported that they were the victims of physical abuse over the previous year [5–7]. In a study conducted at Michigan University, 89% of the violent incidents were by patients, 9% by family members, and 2% by friends of the patient [7]. In a national survey conducted in the United States, 78% of emergency room doctors reported that they were a target of violence at the workplace over the previous year. Of these, 75% were verbal threats, 21% were physical attacks, 5% were confrontations outside of the workplace, and 2% were cases of harassment [8].

Violence towards doctors is not limited to the emergency room. For example, about one third of residents in pediatrics reported attacks by patients or family members over the course of their training [9]. The same study showed that 71% of pediatricians reported that they had not received any training at the workplace during their residency on dealing with attacks by patients or families. Most of them believed that they would benefit from appropriate training on coping with angry patients [9].

The primary reasons for violence directed at the medical staff are long waiting times, dissatisfaction with treatment, a hurtful comment by a staff member, or the influence of drugs and alcohol on the attacker [2].

In a study conducted in the Department of Family Medicine in Beer-Sheva, Israel, that was based on data collected in 2001–2002 and that was published in 2005 [10], we investigated reports of physical and verbal abuse towards doctors in the hospital and the community in the Negev region. In that study, 56% of the participants reported at least one incident of verbal abuse over the previous year and 9% reported that they were exposed to physical abuse at least once over the same period of time. There was no statistically significant difference in exposure to physical or verbal abuse between doctors in the hospital and in the community, but there was a significant difference in the effect of the violence on the doctor’s life. Community-based doctors reported a greater negative effect on their and their family’s quality of life compared to hospital doctors. In that study the major reasons for violent behavior were long waiting times, dissatisfaction with treatment and disagreement with the doctor.

The aim of the present study was to assess trends in violence toward doctors in the hospital and the community in the same geographic region and compare the results with those of the previous study [10].

## Methods

### Study design and selection of study subjects

This was a cross-sectional study of 63 doctors in the Soroka University Medical Center and 82 community doctors (of the approximately 800 and 400 physicians who work in those settings, respectively) who worked for the Clalit Health Services in the southern region. In the few cases when a doctor worked in both the hospital and in the community, the primary place of work was used for statistical analyses.

Data were collected by questionnaire at the morning report in various departments in the hospital and community clinics and at CME (Continuous Medical Education) courses held by the Clalit Health Services.

### Questionnaire

The questionnaire, which was completed anonymously, was identical to the one used in the 2001–2002 study (Additional file 1). It was completed by a convenience sample of doctors attending the aforementioned meetings. We decided to ask doctors to participate in face to face during morning meetings with the aim of achieving a larger response rate than could be expected by a postal distribution of the questionnaires or conducting telephone interviews. We did not conduct personal interviews which achieve the highest response rate, since they are more time consuming, but instead made the request face to face, then waited for physicians to complete it in their own time. The vast majority of doctors who attended the meetings completed the questionnaires. In a few cases the doctors refused to participate because of the pressure of work at the time. It is our impression that the main difference between those who agreed to participate to those who refused was whether or not their clinic or department director gave them free time to do so. When they were given free time the response rate was well over 90%. The questionnaire included socio-demographic data, reports of physical and verbal abuse at the workplace over the previous year, questions about ways they cope with violent incidents, and their attitudes towards the phenomenon.

The inclusion criteria included doctors from the various hospital departments including internal medicine, pediatrics, surgery, gynecology and psychiatry and family doctors and pediatricians in the community who worked for the Clalit Health Services. The exclusion criteria included refusal to participate in the study and doctors with less than 1 year of work experience.

### Statistical analysis

The data were entered into and stored in an Excel worksheet at the Siaal Research Center and statistical analyses were conducted by SPSS (Statistical Package for the Social Sciences**)**. Descriptive analyses were used for socio-demographic characteristics. An initial univariate analysis was used to compare socio-demographic variables and variables related to exposure to violence and ways of coping with it between hospital and community doctors. Chi-square or Fisher’s exact test were used for discrete variables and ANOVA or t-tests for continuous variables. Statistical significance was set at *P* < 0.05 for all analyses.

Both the Ethics Committees of the Soroka University Medical Center (Dec. 12, 2016), and the Ethics Committee for Community-based Studies of the Meir Medical Center (Jan 5, 2017) exempted this study from the requirement to obtain informed consent.

## Results

### Demographic characteristics of the study population

Of the 145 doctors who took part in the study 63 (43.4%) were hospital doctors and 82 (56.6%) worked in the community. There was an equal gender distribution with 72 female and 72 male doctors (missing information for one doctor). The mean age was 44.7 ± 11.5 years. Sixty one (42%) were residents and 84 (58%) were board certified. Among hospital doctors 76.2% were Israeli born compared to 46.9% among community doctors (*P* < 0.0001). Community doctors had a higher mean work seniority than hospital doctors (16.6 ± 12.0 compared to 12.4 ± 10.9 years, *P* = 0.044). The socio-demographic data are presented in Table 1.

**Table 1:** Comparison of demographic characteristics between community and hospital doctors

Variable	Hospital doctors *N* = 63 N (%)	Community doctors *N* = 82 N (%)	All doctors *N* = 145 N (%)	*P*
Gender				
Male	34 (54.0)	37 (46.8)	71 (50.0)	0.499
Female	29 (46.0)	42 (53.2)	71 (50.0)	
Age (Y)				
Mean ± SD	42.7 ± 10.9	46.2 ± 11.7	44.7 ± 11.5	0.074
Range	26–66	28–69	26–69	
Country of birth				
Israel	48 (76.2)	38 (46.9)	86 (59.7)	<0.0001
Abroad	15 (23.8)	43 (53.1)	58 (40.3)	
Board certified				
No	29 (46.0)	32 (39.0)	61 (42.1)	0.403
Yes	34 (54.0)	50 (61.0)	84 (57.9)	
Professional seniority (Y)				
Mean ± SD	12.4 ± 10.9	16.6 ± 12.0	14.8 ± 11.7	0.044
Range	5–26	1–40	1–40	
Teaching involvement				
High involvement	15 (23.8%)	15 (18.8)	30 (21.0)	0.701
Moderate involvement	18 (28.6)	22 (27.5)	40 (28.0)	
Low involvement	17 (27.0)	20 (25.0)	37 (25.9)	
No involvement	13 (20.6)	23 (28.8)	36 (25.2)	

### Comparison of exposure to violence

Fifty nine percent of the doctors reported that they had experienced at least one incident of verbal abuse over the previous year and 9% had experienced physical abuse on the part of patients or their family. Thirty two (50.8%) of the hospital doctors and 40 (49.4%) of the community doctors said that they had been exposed to verbal abuse by patients over the previous year (*P* = 1.000). More hospital doctors were exposed to verbal abuse by family members (*N* = 37, 58.7%) compared to community doctors (*N* = 29, 35.8%) (*P* = 0.007). A similar trend was found for physical abuse by family members, but the results were not statistically significant (7.9% vs. 1.2%, respectively, *P* = 0.086).

The most common reported cause of violence was long waiting time (23.9%), followed by dissatisfaction with treatment (16.9%). There were no significant differences between the two groups in terms of the causes of violence. The only cause of violence that differed in frequency between hospital and community doctors was unjustified requests for medical prescriptions. These were more common among community doctors (15.2%) than hospital doctors (1.6%) (*P* = 0.006).

In most of the study variables there were no differences in exposure to verbal and physical abuse between female and male doctors. When residents and specialists were compared, specialists were more exposed than residents to physical abuse by patients (*P* = 0.013) and the patient’s family members (*P* = 0.039).

### Coping with violent incidents

Eighty six doctors (59.3%) said that they had been exposed at least one time to any type of violence, of these 42 hospital doctors (66.7%) and 44 community doctors (53.7%). In most of the variables analyzed there were no significant differences between hospital and community doctors. The most common method reported for dealing with verbal violence incident was to defuse the situation by communication (43%), followed by alerting a security personal (21.8%). In 14.8% of the cases the doctor chose to ignore the incident and continue working without addressing it at all. Only 7% alerted the police and only 4.2% ended up filing an official complaint to the police.

When asked for the reasons the police were not contacted, the major reasons cited were the feeling that the case wasn’t severe enough to justify a complaint to the police, and acceptance of an apology from the assaulter. However, other reasons were reported, such as fear of the assaulter, pressure from the assaulter or family members to drop charges, and wishing to avoid court sessions.

There was a non-significant trend relating to the negative effect of exposure to violence on other aspects of life than work, with 16 community doctors (42.1%) reporting a negative effect on their private lives compared to nine hospital doctors (22.5%) (*P* = 0.090).

### Attitudes towards workplace violence

In the total study population, 103 doctors (73%) thought that the issue of violence was a major problem for doctors today. Most of them (73.9%) had not undergone a workshop or any other type of training to prepare them to prevent or manage workplace violence. There were no statistically significant differences in this variable between hospital and community doctors, female and male doctors, or residents and specialists.

In describing the attacker, hospital doctors were more likely to describe a family member (69.6%) as the perpetrator compared to 43.1% of community doctors (*P* = 0.004). Community doctors were more likely to describe the attacker as having a known psychiatric disorder (*P* = 0.002), being a drug addict or alcoholic (*P* = 0.012), or a smoker (*P* = 0.007). Female doctors (66.7%) were more likely to describe the attacker as a family member than male doctors (44.6%) (*P* = 0.019) and were more likely to state that the attacker had a history of previous violent outbursts (*P* = 0.031).

In response to an open question on how to deal better with the phenomenon of workplace violence, 63.4% of the doctors said that prevention and security measures should be strengthened, 34.1% thought that patient education and advocacy of the issue was needed, 26.8% were in favor of appropriate training for the medical staff to improve coping with these incidents, and 26.8% thought that the solution should be improving communication between the doctor and the patient. Additional solutions that were suggested were improved availability of healthcare services with better treatment conditions, support from the administration and more teamwork among the staff members, and physical adjustment of the workplace (such as panic buttons). Data on proposed solutions appear in Table 2.

**Table 2:** Can the phenomenon of violence be handled?

How in your opinion can violence be handled in a better way (more than one answer is possible)?	N (%)
Increased enforcement and deterrence	52 (63.4)
Education of patients	28 (34.1)
Staff training	22 (26.8)
Better communication between doctors and patients	22 (26.8)
Improved availability and quality of care	17 (20.7)
Teamwork	13 (15.9)
Adjustment of the work environment	6 (7.3)

### Comparison with the former study (2005 vs. 2018)

In terms of overall exposure to verbal or physical violence there were no significant differences between the present study and the previous one by Carmi-Iluz et al. [10]. Fifty nine percent of all doctors in the current study suffered from verbal abuse, which is similar to the 56% who reported incidents of verbal violence in the previous study (*P* = 0.999). Nine percent suffered physical abuse, the same as in the previous study (*P* = 0.997). No significant differences were seen when exposure to violence was compared by subgroups such as hospital physicians compared to community physicians, or whether the attacker was a patient or a family member.

The percentage of physicians who reported receiving training on the prevention and management of workplace violence (26.1%) improved compared to the 17% who reported similar training in the previous study (*P* = 0.047).

Table 3 presents comparative details between the two studies.

**Table 3:** Comparison of main variables with the previous study (2005 vs. 2018)

	2005 (*N* = 177) N (%)	2018 (*N* = 144) N (%)	*P*
Any verbal abuse			
Yes	99 (56)	85 (59)	0.999
No	78 (44)	59 (41)	
Any physical abuse			
Yes	16 (9)	13 (9)	0.997
No	161 (91)	131 (91)	
Did you attend a workshop on coping with violence?			
Yes	30 (17)	36 (26.1)	0.047
No	147 (83)	105 (73.9)	

## Discussion

In this study we found that 59% of the doctors reported having been exposed to at least one act of verbal abuse over the previous year and 9% reported physical abuse. The findings in the present study are similar to those of Carmi-Iluz et al. [10], with the same rate of exposure to physical abuse at the workplace, and a slight, non-significant increase in exposure to verbal abuse from 56 to 59%.

We were surprised by the similarity in the level of exposure to violence between the two studies. According to data collected recently by the Israel Ministry of Health, 3500 acts of violence are reported in the healthcare system per year [11], in contrast to 1440 cases in 2001 [10]. Since most acts of violence end without filing of complaints with the police, it is reasonable to assume that, in reality, the actual number is much higher. We do not know why the results of our study do not reflect this increase in violent incidents, we can speculate that violent assaults may not have increased as much over the years as it seems, but awareness has increased, resulting in a larger number of assaults being reported to the Ministry of Health and in the media.

It is interesting to note that although it is commonly believed that hospital-based cases of violence are more frequent due to the higher levels of stress that accompany a visit to the hospital, in the end both hospital based and community based doctors are exposed to a similar number of violent attacks. This similarity may be explained by the fact that the number one reason for a violent attack, as reported by physicians in this study, is long waiting times. As the Israeli population increases both in numbers and in age, the wait to see a doctor gets longer in both the emergency room and the local clinic. It is noteworthy that this similarity is stronger for verbal abuse. When asked about exposure to verbal abuse by patients themselves, there was no difference between hospital and community doctors, (*P* = 1.000). However, when asked about exposure to physical violence by both patients and their family members, 14.3% of hospital physicians reported at least one incident in comparison with 4.9% of community doctors, a difference close to statistical significance (*P* = 0.077). Had the difference reached statistical significance, it would have been justified to analyze the reasons. Since it was not, we will briefly mention two factors that might be related to the non-significant difference found: (1) physical abuse by patients’ family members, which affected hospital doctors more and (2) the higher level of stress that could have exacerbated any confrontation.

Our assumption that the percentage of doctors who participated in a workshop or any other form of training to cope with violence at the workplace would have increased was corroborated with 26.1% in the present study compared to 17% in the earlier study [10] (*P* = 0.047). Despite this improvement, one can see that most doctors still did not undergo any form of training to cope with acts of violence, even though about 92% believed that violence represents a moderate to severe problem for doctors today.

Most cases of violent attacks were resolved by the physician who was attacked without intervention by a third party such as the institute’s security personnel or the police. While this finding strengthens our assumption that the number of violent incidents reported by the Israeli Ministry of Health is, in fact, an underestimation, It also emphasizes the importance of formal training on ways that physicians can cope with and defuse violent situations since, in most cases, they are the ones dealing with the incident without outside assistance.

In the present study we found that a higher percentage of hospital doctors than community doctors were exposed to verbal abuse by patients’ family members (*P* = 0.007) and a similar trend, although not statistically significant, was seen for physical abuse (*P* = 0.086). These findings are consistent with those of a previous study by Carmi-Iluz et al. [10]. The difference can be explained in that hospital doctors are exposed, during the course of their daily work, to a larger number of family members who accompany patients in the emergency room or during hospitalization, while family doctors see a patient population that are more likely to come to the doctor alone. Another possible explanation, as mentioned by Carmi-Iluz et al., is that family members may feel less constrained to confront hospital physicians, whom they don’t know, than family physicians with whom they may have an ongoing relationship.

Workplace violence has ramifications beyond immediate physical damage. In a study conducted in China, workplace violence was associated with a feeling of increased psychological stress, burnout, and depression, all of which had a negative effect on the quality of sleep and health in general [12]. In the present study 42.1% of community doctors reported that they felt that violence had a negative effect on other aspects of their lives, compared to 22.5% of hospital doctors (*P* = 0.090). This trend, although not statistically significant, is consistent with findings in the study by Carmi-Iluz et al. [10] in which a statistically significant difference on this issue was found. This variability could be explained by the ongoing relationship of community doctors with violent patients in contrast with the short and transient contact that hospital doctors have with most of their patients. In light of these similar findings it is possible that if we had a larger sample size our finding would also have reached statistical significance.

The most common presumed cause of the outbreak of violence as reported in our study was a long waiting time (23.9%). This result is consistent with those of previous studies on this issue, including the study by Carmi-Iluz et al. [10] and a study from 2015 that was conducted in the Palestinian Authority [13]. This association highlights yet another undesired outcome of inadequate working conditions, in which a small number of physicians care for a large number of patients, and provides yet another argument to increase the number of physicians in Israel, both in hospitals and in outpatient clinics. However, the results of an Israeli study published in 2017 [14] that assessed the causes of workplace violence among doctors, nurses, and security personnel, showed that only 10% of the participants considered a long waiting time as the cause of violence, compared to 39% who considered staff behavior as a leading cause of violence. In that study, 35% of the staff thought that their behavior contributed to the most serious acts of violence that they were involved in. This cause is cited much less frequently in the literature. It makes sense that doctors would find it more difficult to provide evidence of their own role in conflicts with patients, especially in cases in which they were the victims of physical abuse. While there is no justification for violent behavior, it is appropriate to conduct an in-depth investigation of the types of behavior that expose doctors to increased danger of violence and to make recommendations as to how to reduce the number of violent events.

Further research should be conducted on ways to train physicians to cope with violent incidents. The increased number of physicians who participated in such training is encouraging, but we did not collect any information on the quality and content of that training, or its efficacy.

In conclusion, several steps should be taken so that workplace violence in the healthcare system be properly addressed and reduced. These include training physicians to cope with such incidents, strengthening collaborations with law enforcement authorities, and reducing waiting times in the hospital and in community clinics.

### Study limitations

Our study has several limitations. First, it is based on a convenience sample of doctors in clinics and/or at staff meetings during the workday. The study was conducted in a specific geographical region in Israel, so its results may not be generalizable to all of Israel or other countries in the world. The data were collected retrospectively and were based on recollection of subjective experiences and not prospectively, which might have enabled us to obtain the perspective of the attacker as well. There are several characteristics of violence towards doctors that were not related to directly in this study. For example, we did not include questions relating to sexual harassment as a type of violence that doctors deal with that has been reported in several other studies in this field [13, 15]. Similarly, focusing on the comparison between community and hospital doctors did not allow us to go more deeply into the unique characteristics of each group, for example the increased exposure of emergency room doctors and psychiatrists compared to other hospital doctors, or the unique risk facing community doctors who make house calls [1, 16].

## Conclusion

Workplace violence is a major issue which affects hospital and community physicians alike. Although more doctors participate in workshops to cope with acts of violence, the majority of doctors have still not undergone any form of training in this field. Such training is crucial as most cases of violence are handled by physicians themselves without outside help. Reducing waiting times and physician workloads is needed to minimize incidents of violence and provide better treatment in general.

We believe that the results of this study can contribute to existing knowledge on violence towards hospital and community doctors by patients and their family members. New solutions should be sought to reduce the extent of violence and to deal more effectively with this worrisome phenomenon.

## Supplementary information

**Additional file 1.** Questionnaire on violence towards doctors in the Negev.

## Data Availability

The data and material are available, depending on the approval of the Ethics Committees of the Soroka University Medical Center and the Meir Medical Center for community-based studies, and the approval of the institutions (Soroka Medical Center and Clalit Health Services). The datasets used/or analyzed during the current study are available from the corresponding author on a reasonable request.

## Abbreviations

CME: Continuous Medical Education
SPSS: Statistical Package for the Social Sciences
