# Supplementary material for: Manifestations of verbal and physical violence towards doctors: a comparison between hospital and community doctors
Source: BMC Health Serv Res. 2019 Nov 26;19:888. doi: 10.1186/s12913-019-4700-2 (PMC6880572; doi:10.1186/s12913-019-4700-2)
Supplement: Supplementary file 1 — Additional file 1. Questionnaire on violence towards doctors in the Negev. [file 12913_2019_4700_MOESM1_ESM.docx]

Additional file1: Questionnaire on violence towards doctors in the Negev

Dear doctor,

Based on studies conducted in Israel and throughout the world many doctors have faced verbal and even physical abuse in the course of their work. Verbal abuse can be defined as an attempt to hurt someone else by means of blunt words, curses, aggressive speech, threats or any other inappropriate expression that does not lead to actual physical damage. Physical abuse can be defined as any form of physical aggression against another person, manifested as a physical attack.

Within the framework of the Research Experience project towards an MD degree we are collecting data on your personal experience with verbal and physical abuse towards doctors over the course of the previous year. We are asking you to consent to complete this questionnaire.

The questionnaire is anonymous. It is formulated in the male gender but is appropriate for males and females alike.

We thank you for your cooperation.

Tamar Nevo, medical student

Socio-demographic data

1. Sex
2. Male
3. Female
4. Year of birth ______
5. Country of birth
6. Israel
7. Eastern Europe
8. Western Europe
9. Former Soviet Union
10. North Africa
11. North America
12. Other
13. Residence
14. Beer-Sheva
15. Satellite communities of Beer-Sheva
16. Kibbutz
17. Moshav
18. Bedouin settlement
19. Other
20. Place of work
21. Hospital
22. Community clinic
23. Country of graduation from medical school
24. Israel
25. Eastern Europe
26. Western Europe
27. Former Soviet Union
28. North America
29. Other
30. Specialization
31. Internal medicine
32. Surgery
33. Pediatrics
34. Psychiatry
35. Family medicine
36. General practitioner
37. Have you finished your residency training?
38. No
39. Yes
40. Work status
41. Department/clinic director
42. Unit director
43. Board certified
44. Resident
45. General practitioner
46. What is your primary clinic setting?
47. Soroka University Medical Center – Internal Medicine department
48. Soroka University Medical Center – Surgical department
49. Soroka University Medical Center – Pediatrics department
50. Soroka University Medical Center – Psychiatry department
51. Soroka University Medical Center – Emergency room
52. Clinic outside of Beer-Sheva – Jewish sector
53. Clinic outside of Beer-Sheva – Bedouin sector
54. Clinic within Beer-Sheva – Jewish sector
55. Clinic within Beer-Sheva – Bedouin sector
56. Other ________________________________
57. Years of seniority as family doctor (since graduation from medical school ___________________
58. The extent of your involvement in teaching in the faculty?
    1. Very strong
    2. Moderate
    3. Low
    4. Not involved at all

Questions on violence

1. Did you experience verbal abuse from your patients over the previous year?
   1. No
   2. Yes, if yes, how many times? ____________________
2. Did you experience verbal abuse from your patients’ family members over the previous year?
   1. No
   2. Yes, if yes, how many times? ____________________
3. Did you experience physical abuse from your patients over the previous year?
   1. No
   2. Yes, if yes, how many times? ____________________
4. Did you experience physical abuse from your patients’ family members over the previous year?
   1. No
   2. Yes, if yes, how many times? ____________________
5. In the case of physical abuse, how were you attacked?
   1. Blows from a hand
   2. Throwing of an object
   3. Threatened with a knife
   4. Threatened with a weapon
   5. I was not attacked
   6. Other (specify) ___________
6. Where did the event occur?

*In cases of verbal abuse*:

- 1. In the department
  2. In the emergency room
  3. In the doctors’ room
  4. Not relevant

*In cases of physical abuse*:

- 1. In the department
  2. In the emergency room
  3. In the doctors’ room
  4. Not relevant

1. Did you feel that your health was endangered because of physical abuse over the previous year?
   1. No
   2. Yes, to a slight degree
   3. Yes, to a moderate degree
   4. Yes, to a great degree
2. Have you felt that your life was endangered because of physical abuse over the previous year at the workplace?
   1. No
   2. Yes, to a slight degree
   3. Yes, to a moderate degree
   4. Yes, to a great degree
3. When did these events take place?
   1. In the morning hours or at noon
   2. In the afternoon
   3. In the evening
   4. At night
   5. There were no events of this nature
4. On the last occasion that this occurred, what was the reason for the outbreak of violence?
   1. No apparent reason
   2. Long waiting time
   3. Dissatisfaction with treatment
   4. Disagreement with the doctor
   5. Reaction to a sentence that the attacker did not like
   6. Delivering of bad news
   7. Negatively biased views of the doctor or the hospital
   8. Monetary reasons
   9. Unjustified request for a medical certificate
   10. Other __________________-
5. How did you react to the act of violence towards you? (More than one response possible)
   1. Ignored it
   2. Verbal response
   3. Summoned security
   4. Summoned the police
   5. Filed a complaint with the police
   6. Other _________________
   7. No event of this type occurred

If not act of violence occurred, please skip to question 33

1. How did you feel during the event?

Please rank each item on a scale of 1-8, with 1 as the lowest level of intensity and 8 the highest level of intensity

- 1. Threatened __________
  2. Anxiety __________
  3. Impotence __________
  4. Humiliation __________
  5. Anger ___________
  6. Apathy ____________
  7. In control of the situation __________

1. Why did you react as you did?
   1. Did not want to cause a disturbance
   2. Threats by the patient or the patient’s family
   3. Fear of physical injury
   4. Other ____________________
2. How did the event end from your perspective?
   1. Without injury
   2. With a negative emotional effect
   3. Physical injury with a negative emotional effect
   4. Other __________________­
3. Did the event have a negative effect on you in areas other than work, for example in terms of the family or your quality of life?
   1. No
   2. Yes
4. For how long did the event affect you?
   1. That same day
   2. Up to one month
   3. More than one month
5. How was the event handled by the hospital or in the community?
   1. It wasn’t handled
   2. Security personnel removed the attacker
   3. The hospital/the clinic director did not approve filing of a complaint
   4. It is being investigated by the police
   5. The attacker was indicted
   6. The case was closed for lack of public interest
6. Were you satisfied with the way the event was handled?
   1. No
   2. Yes
   3. Somewhat
7. In the event of abuse (verbal or physical) where no complaint was filed, what were the reasons for not filing one.
   1. Fear of the patient or the family member
   2. Pressure by the patient or the family member to rescind the complaint
   3. Apology by the attacker
   4. Satisfaction with the way security handled the event
   5. Lack of will to go to court
   6. The hospital/the clinic director did not approve filing of a complaint
   7. An event that was not severe enough to justify turning to the police
   8. Other ___________________
8. How would you handle a similar case today?
   1. Ignore it
   2. Verbal response
   3. Summon security
   4. Summon the police
   5. File a complaint with the police
   6. Other ____________
9. Do you think that this issue of violence is a problem for doctors today?
   1. Yes, to a great degree
   2. Yes, to a moderate degree
   3. Yes, somewhat
   4. No
10. Did you participate in a workshop or any other form of training to cope better with acts of violence at the workplace?
    1. No
    2. Yes
11. How would you characterize individuals who have violent tendencies **based on your experience**? (More than one response is possible)
    1. Male
    2. Female
    3. Young, up to 40 years of age
    4. Older, more than 40 years of age
    5. Israeli born
    6. New immigrant
    7. Jewish – Ashkenazic origin
    8. Jewish – Sephardic origin
    9. Bedouin
    10. Patient
    11. Family member of the patient
    12. Good socio-economic status
    13. Poor socio-economic status
    14. Low education level
    15. Academician
    16. Known mental disorder
    17. Addicted to drugs or alcohol
    18. Smoker
    19. With a prior history of violence
    20. Criminal record
    21. Other ________________
    22. Other ________________
    23. Other ________________
12. In which ways do you think that violence could be handled better?
    1. _________________________________________
    2. __________________________________________
    3. __________________________________________
    4. __________________________________________
    5. __________________________________________
    6. __________________________________________
    7. __________________________________________

Thank you for your cooperation!
